# 沉默PPAR-γ通过上调bcl-2表达抑制A549细胞凋亡

**DOI:** 10.3779/j.issn.1009-3419.2013.03.02

**Published:** 2013-03-20

**Authors:** 靖宇 杨

**Affiliations:** 1 300070 天津，天津医科大学研究生院 Graduate School of Tianjin Medical University, Tianjin 300070, China; 2 300051 天津，天津胸科医院 Tianjin Chest Hospital, Tianjin 300051, China

**Keywords:** PPAR-γ, bcl-2, 细胞凋亡, 肺肿瘤, PPAR-γ, Bcl-2, Apoptosis, Lung neoplasms

## Abstract

**背景与目的:**

肺癌耐药是肺癌患者死亡的主要原因，PPAR-γ可促进细胞凋亡，逆转肺癌耐药。本实验旨在探讨PPAR-γ表达下调对人肺癌A549顺铂耐受性和细胞凋亡的影响。

**方法:**

构建siRNA沉默PPAR-γ的A549细胞系[A549/PPAR-γ(-)]，应用MTT法检测顺铂对PPAR-γ沉默A549细胞增殖的影响，应用流式细胞术检测顺铂对PPAR-γ沉默A549细胞周期的影响，Western blot法检测磷酸化Akt（p-Akt）、caspase-3和bcl-2/bax的变化，最后以RT-PCR检测bcl-2的转录水平。

**结果:**

成功构建出两个A549/PPAR-γ(-)细胞克隆，经RT-PCR和Western blot检测其PPAR-γ的水平明显下降。PPAR-γ沉默后，两个克隆A549细胞对顺铂的耐受性分别增加了1.29倍和1.60倍，肿瘤细胞的凋亡减少。Western blot检测显示Akt的磷酸化水平和bcl-2/bax水平升高，caspase-3表达降低，RT-PCR进一步显示bcl-2的转录水平升高。

**结论:**

抑制A549中PPAR-γ的表达后，肿瘤细胞获得对顺铂药物更高的耐受性，其机制与升高Akt磷酸化水平和bcl-2表达水平，抑制细胞凋亡有关。PPAR-γ下调是临床肿瘤产生耐药性的可能机制之一。

## Abstract (en)

**Background and objective:**

Drug resistance is the one of primary causes of death in patients with lung cancer, PPAR-γ could induce the apoptosis and reverse drug resistance. The aim of this study is to investigate the expression of PPAR-γ on cisplatin sensitivity and apoptosis response of human lung cancer cell line A549.

**Methods:**

Reconstruction of PPAR-γ silencing A549 cells (A549/PPAR-γ(-)) by siRNA. MTT assay was employed to determine the effect of cisplatin on the proliferation of A549/PPAR-γ(-), flow cytometry to determine the effect of cisplatin on the cell apoptosis, Western blot to determine the change of phosphorylation of Akt, caspase-3 and expression of bcl-2/bax. Finally, RT-PCR was employed to determine the transcriptional level of bcl-2.

**Results:**

Two PPAR-γ silencing A549 cell clones were established successfully, and the expression of PPAR-γ was downregulated significantly as confirmed by RT-PCR and Western blot. After PPAR-γ silencing, the resistance of these two A549 clones to cisplatin was increased by 1.29-fold and 1.60-fold respectively. Flow cytometry showed that the apoptosis rate was decreased, and Western Blot showed that the phosphorylation of Akt and expression of bcl-2/bax were upregulated, caspase-3 was downregulated. Finally, RT-PCR showed that the transcriptional level of bcl-2 was upregulated as well.

**Conclusion:**

Downregulation of PPAR-γ in A549 cells led to increase of cisplatin resistance. One of the mechanisms was upregulatin of phosphorylation of Akt and expression of bcl-2, which inhibited the apoptosis of cells. The downregulation of PPAR-γ is a possible mechanism that leads to the clinical drug resistance of cancer.

肺癌是癌症引发死亡的第一大原因，2008年在全球范围内大约造成1, 380, 000例死亡^[1]^。根据组织学分类，肺癌可分为非小细胞肺癌和小细胞肺癌，其中前者占据肺癌的80%^[2]^。肺癌预后很差，目前化疗仍然是晚期疾病治疗的主要手段。铂类药物，包括顺铂和卡铂，是此类疾病化疗最常用的药物，肿瘤细胞对铂类药物产生耐药性是临床治疗失败的主要原因^[3]^，克服耐药性是提高临床治疗效果的迫切需要。铂类药物与DNA的碱基结合，干扰DNA合成，抑制肿瘤细胞增殖并诱发细胞凋亡。肿瘤细胞耐受铂类药物的机制主要为两方面，一方面过表达多药耐药基因（multidrug resistance genes, MRG）以减少药物在细胞内的蓄积，另一方面提高肿瘤细胞抗凋亡能力。对这两种机制涉及的信号通路做深入研究并寻找克服耐药性的方法一直是癌症研究的重点。已发现的机制包括Src激酶活性升高诱导MDR1和LRP过表达^[4]^，PI3K/Akt信号通路异常活跃导致肺癌转移和耐药^[5]^。

PPAR-γ（peroxisome proliferator-activated receptor gamma）是一种Ⅱ型核受体，在人类细胞中由*PPARG*基因编码。PPAR-γ的基本功能包括调节脂肪酸的储存和糖代谢，因此临床上它与肥胖、糖尿病和动脉粥样硬化等代谢疾病密切相关^[6]^。同时，PPAR-γ还与癌症密切相关。临床前研究^[7]^发现，配体激活PPAR-γ可控制肿瘤的生长，促进细胞凋亡，抑制肿瘤转移。因此，*PPARG*被认为是一个抑癌基因，靶向PPAR-γ是治疗肿瘤的一个潜在有效的手段。然而，PPAR-γ与肿瘤耐药的关系尚未得到充分的研究。本文对PPAR-γ沉默后A549顺铂耐药性的变化进行研究，并对其中的分子机制进行初步的研究。

## 1 材料与方法

### 1.1 主要试剂与仪器

人非小细胞肺癌细胞系A549购自中国科学院上海生命科学研究院生物化学与细胞生物学研究所；细胞培养基购自Gibco；PPAR-γ siRNA质粒及对照siRNA质粒均购自Santa Cruz生物技术公司；MTT试剂、RT-PCR试剂盒购自Promega公司；凋亡检测试剂盒购自BD公司；顺铂购自Sigma公司；抗PPAR-γ、Akt、p-Akt、caspase-3、bcl-2和bax单抗购自Santa Cruz公司；ECL免疫印迹底物试剂盒购自Millipore；流式细胞仪：BD公司，酶标仪：Thermo，PCR仪：Thermo。

### 1.2 细胞与细胞培养

A549培养于10 cm培养皿，37 ℃、5% CO_2_、饱和湿度的培养箱中，培养基为90% F-12K，10%胎牛血清（FBS）。0.25%胰酶-EDTA消化传代，所有试验均采用对数生长期细胞。

### 1.3 PPAR-γ siRNA质粒转染A549细胞并筛选稳定转染的细胞克隆

A549培养于6孔板中，按照试剂盒说明书进行转染。经优化，按照1 μg siRNA Plasmid：1 μL siRNA Plasmid Transfection Reagent的比例配制转染试剂。当细胞融合程度达到60%-70%时，用2 mL siRNA Transfection Medium洗涤细胞2次，每孔加入0.8 mL siRNA Transfection Medium和0.2 mL上述转染试剂（含1 μg质粒），继续培养6 h后，加入1 mL含20% FBS的培养基，继续培养48 h，吸走培养基，加入含有4 μg/mL嘌呤霉素的选择培养基。在该浓度的嘌呤霉素下，未经转染的细胞在4天后即死亡。每隔3-4天更换选择培养基，直至得到克隆生长的细胞。

### 1.4 MTT法检测顺铂对细胞生长的作用

取对数生长期的A549以及克隆细胞，分别以4×10^4^个/mL接种到96孔微孔板中，100 μL/孔，培养过夜使细胞贴壁。向对应试验孔加入不同浓度的顺铂，继续培养72 h，吸去培养基，加入100 μL含0.5 mg/mL MTT的F-12K，继续培养4 h。吸去MTT，加入100 μL DMSO，使MTT结晶完全溶解，最后用酶标仪测定490 nm波长下的OD值，并计算药物对细胞的抑制率。抑制率＝（1-实验组OD值/对照组OD值）×100%。以顺铂浓度的对数为横坐标，抑制率为纵坐标作图并拟合抑制曲线，50%抑制率所对应的化合物浓度即为IC_50_值。

### 1.5 流式细胞术检测细胞凋亡

取对数生长期的A549和A549/PPAR-γ(-)细胞，分别加入10 μmol/L顺铂，继续于培养箱内培养24 h，然后收集上清及贴壁的细胞。调整细胞浓度至1×10^6^/mL，用凋亡检测试剂盒染色，最后用流式细胞仪检测细胞。该试剂盒通过Annexin V与外翻的磷脂酰丝氨酸结合，PI与细胞核结合，检测凋亡细胞。

### 1.6 Western blot法检测细胞PPAR-γ、p-Akt、caspase-3和bcl-2/bax的表达

取对数生长期的A549和A549/PPAR-γ(-)细胞，收集细胞裂解提取蛋白。BCA法测定细胞裂解物的蛋白含量，取等量蛋白质以12%SDS-PAGE法分离并转移至PVDF膜上，以相应的单克隆抗体室温孵育4 h以检测目标蛋白。洗去一抗，以HRP连接的二抗室温孵育2 h，洗涤后以ECL试剂盒显示免疫反应条带。β-actin作为内参。

### 1.7 RT-PCR检测细胞中*PPARG*和*bcl*-2基因的mRNA水平

取对数生长期的A549和A549/PPAR-γ(-)细胞，用Trizol法提取各组总RNA，用Real-Time PCR试剂盒进行逆转录得到cDNA。PPAR-γ上游引物序列：5'-ACTGTCGGTTTCAGAAGTGC-3'，下游引物序列：5'-ATGGACACCATACTTGAGC-3'；bcl-2上游引物序列：5′-ACGGGGTGAACTGGGGGAGGA-3′，下游引物序列：5′-TGTTTGGGGCAGGCATGTTGACTT-3′；以β-actin为内参，上游引物序列：5'-TCCTGTGGCATCCACGAAACT-3'，下游引物序列：5'-GAAGCATTTGCGGTGGACGAT-3'。94 ℃变性3 min后，按下述条件扩增40个循环：95 ℃、5 s，65 ℃、35 s，72 ℃、60 s，循环后72 ℃延伸5 min。

### 1.8 数据统计

每组实验均重复3次，实验数据以Mean±SD表示，使用SPSS 13.0软件进行分析。采用单因素方差分析（*One-way* *ANOVA*）进行比较，以*P* < 0.05表示差异具有统计学意义。

## 2 结果

### 2.1

经siRNA转染的A549细胞克隆表达更低的PPAR-γ PPAR-γ siRNA质粒或对照siRNA质粒转染的A549细胞，在含有嘌呤霉素的选择培养基中筛选并克隆化培养。只有成功转入质粒的细胞方能在选择培养基中生长。经过克隆增殖后，收集克隆细胞以RT-PCR和Western blot检测PPAR-γ的表达水平，验证siRNA沉默效果，最终确定克隆1组和克隆2组。两个克隆间的沉默水平不同，命名为沉默组1和沉默组2（siRNA-1 group和siRNA-2 group），用于后续研究，如图 1所示，两例克隆细胞的PPAR-γ蛋白表达量均明显低于母细胞A549（control group）和空载体阴性对照组（negative group），沉默组PPAR-γ mRNA表达分别为母细胞组的42.6%±2.22%（*P* < 0.000, 1）和28.5%±1.63%（*P* < 0.000, 1），对照siRNA质粒转染克隆A549细胞的PPAR-γ表达水平保持不变。

**1:**
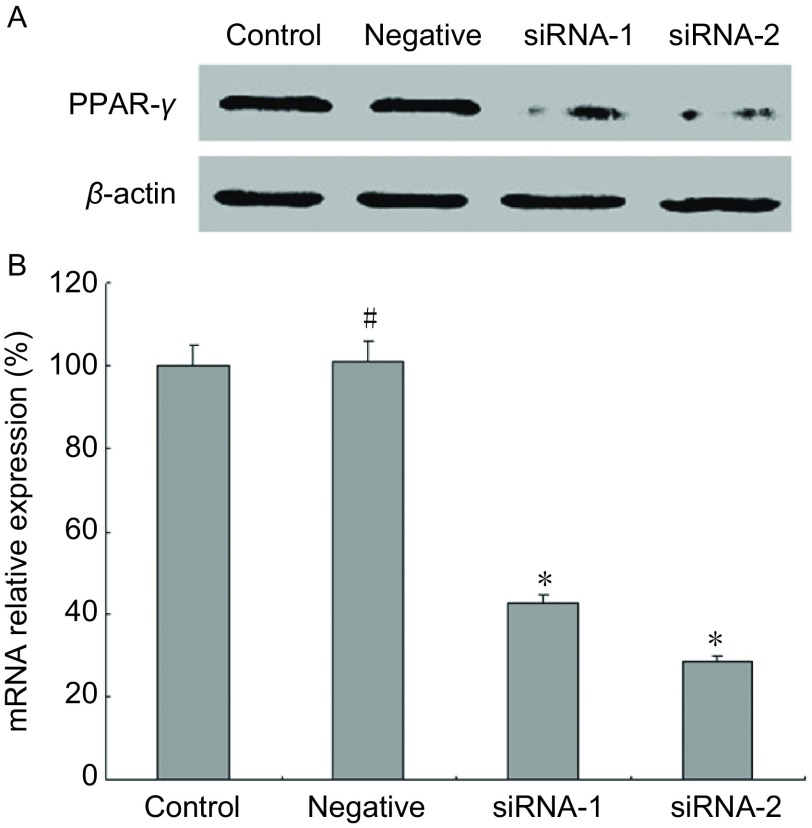
PPAR-*γ*沉默表达A549细胞系的构建。^#^*P*>0.05，^*^P < 0.05 The construction of stably PPAR-*γ* silencing expression A549 cell lines. The expression of PPAR-γ in A549 cell lines was measured by Western blot (A) and real time PCR (B). ^#^*P*>0.05, ^*^P < 0.05

### 2.2 PPAR-γ表达沉默提高细胞对顺铂的耐受性

以母系A549细胞，对照siRNA和PPAR-γ siRNA转染的细胞为研究对象，通过MTT法分别检测细胞对顺铂的敏感性（图 2）。试验结果显示，对照siRNA转染的细胞对顺铂的敏感性，IC_50_为（11.4±0.82）μmol/L，母细胞A549为IC_50_为（12.3±0.78）μmol/L，相比未发生明显变化，而PPAR-γ沉默后的细胞对顺铂的耐受性增加，表现为IC_50_值的升高，沉默组1的IC_50_为（14.7±0.97）μmol/L，沉默组2的IC_50_为（18.2±1.08）μmol/L。

**2:**
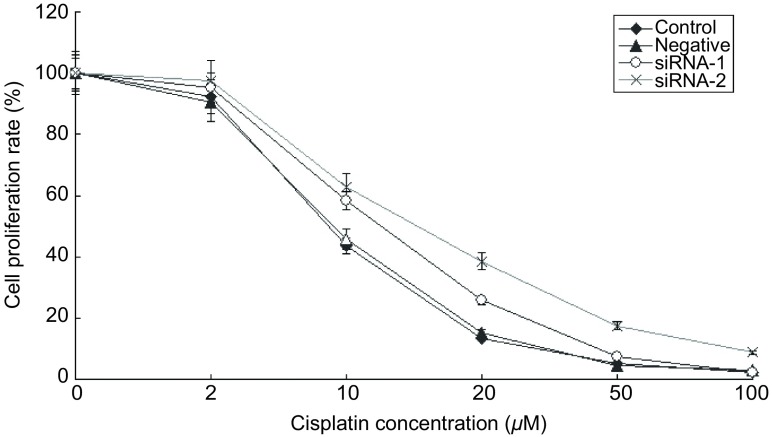
PPAR-*γ*对A549细胞药物敏感性的抑制作用 Inhibition effect of PPAR-*γ* on A549 cells drug sensitivity

### 2.3 PPAR-γ表达沉默抑制细胞凋亡

为寻找PPAR-γ沉默后细胞对顺铂耐受性提高的原因，分析对比了顺铂处理后细胞凋亡的比例。流式细胞术检测结果显示，在顺铂的作用下，A549的凋亡细胞明显可见。母细胞组凋亡率为43.2%±2.42%，经PPAR-γ沉默的沉默组1和组2其凋亡细胞比例分别为32.5%±1.73%（*P*=0.003, 4）和25.1%±1.34%（*P*=0.000, 3），明显低于母细胞组（图 3），这一结果与MTT结果一致，说明细胞对顺铂耐受的提高与细胞抗凋亡能力的提高有关。

**3:**
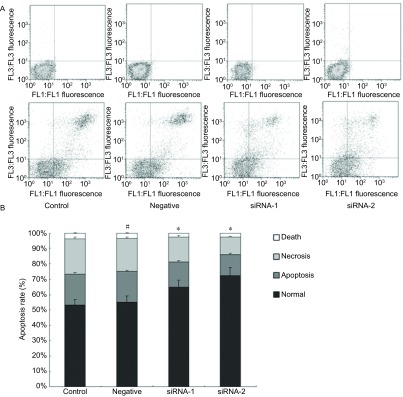
PPAR-*γ*对A549细胞凋亡的抑制作用。^#^*P*>0.05，^*^P < 0.05 Inhibition effect of PPAR-*γ* on A549 cells apoptosis. ^#^*P*>0.05, ^*^P < 0.05

### 2.4 PPAR-γ表达沉默抑制细胞凋亡与Akt磷酸化、caspase-3和bcl-2/bax表达水平上调有关

为进一步探索PPAR-γ调控细胞凋亡的机制，通过Western blot检测细胞在顺铂处理后Akt磷酸化、caspase-3和bcl-2/bax表达水平。与母细胞相比，沉默组1和沉默组2细胞中Akt磷酸化水平和bcl-2/bax的表达水平明显上升，caspase-3表达水平明显下降（图 4）。

**4:**
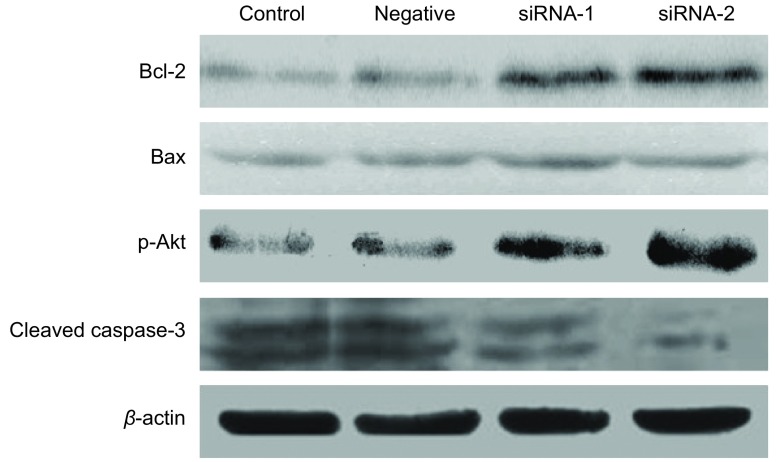
PPAR-*γ*对A549细胞凋亡相关基因蛋白表达的影响 The effect of PPAR-*γ* on protein expression of apoptosis-relative gene in A549 cell lines

### 2.5 PPAR-γ对bcl-2表达的调控源自基因转录水平

RT-PCR分析显示，相比对照细胞，A549/PPAR-γ(-)在顺铂处理后，bcl-2 mRNA水平明显上调，分别为母细胞组的156.2%±11.23%（*P* < 0.000, 1）和188.4%±13.46%（*P* < 0.000, 1）（图 5），这一结果与Western blot的结果一致，说明PPAR-γ对bcl-2表达的调控是通过转录水平实现的。

**5:**
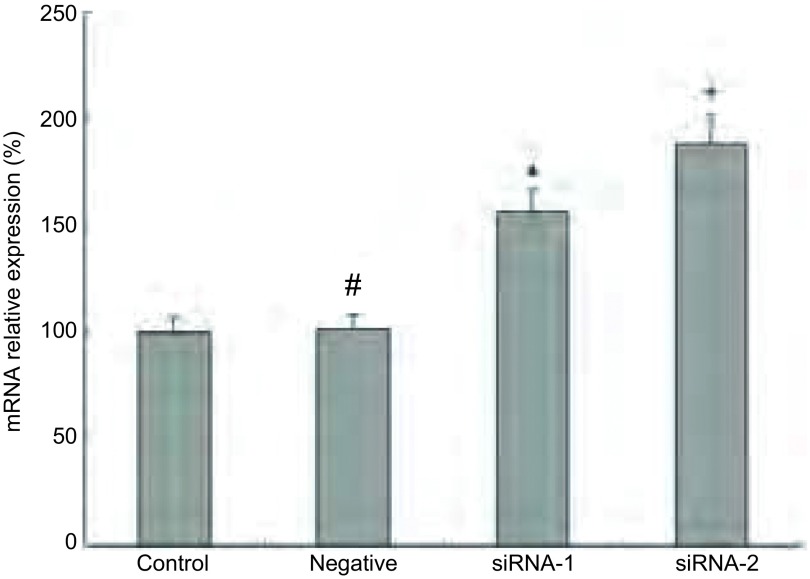
PPAR-*γ*对A549细胞bcl-2 mRNA表达的影响。^#^*P*>0.05，^*^P < 0.05 The effect of PPAR-*γ* on mRNA expression in A549 cell lines. *β*-actin was used as an internal. ^#^*P*>0.05, ^*^P < 0.05

## 3 讨论

肺癌是全球癌症的第一原因，也是癌症的第一死因。铂类药物作为肺癌的一线化疗药物，在疾病的治疗中表现出一定的效果，但随着治疗时间的推移，肿瘤往往产生耐药性，导致治疗失败。深入研究肿瘤对铂类化疗药物产生耐药的分子机制，并寻找新的可运用于临床的治疗策略，是提高患者临床受益的迫切需要。

肿瘤细胞对化疗药物产生耐受性的最常见机制有两种^[8]^，一种是过表达特定的多药耐药蛋白，如MDR1、MRP1等，这些蛋白具有跨膜转运的功能，可以消耗ATP的方式将药物从细胞内泵出细胞外，降低药物在细胞内蓄积和药靶部位的有效浓度；另一种机制则是针对药物诱导细胞凋亡的作用特点，肿瘤细胞的分子调控网络发生一系列的变化，导致细胞抗凋亡能力提高，凋亡减少。

PPAR-γ属于PPARs家族的一员，该家族为核转录因子，在与相应的配体结合后介导目标基因的表达或者抑制^[6]^。PPAR-γ高表达于脂肪组织中，它是脂肪细胞分化的主要调控因子^[9, 10]^。同时，PPAR-γ也表达于其它组织中，例如乳腺、结肠、肺、卵巢、前列腺和甲状腺中^[11]^。研究进一步发现PPAR-γ与癌症存在特定关系。在乳腺癌中，PPAR-γ高表达的患者具有更好的无疾病生存期^[12]^。同样地，PPAR-γ也表达于小细胞和非小细胞肺癌中^[13]^。在非小细胞肺癌细胞系中，配体诱导PPAR-γ活化可诱导细胞生长停滞，促进肿瘤细胞分化并诱导凋亡^[14-16]^。然而，PPAR-γ在肿瘤耐药中的作用还没有得到充分的研究。

根据其在肿瘤细胞中的作用，我们推测PPAR-γ的下调可能是肿瘤细胞耐药的机制之一。因此，本研究中我们首先构建了质粒转染的可稳定下调PPAR-γ的非小细胞肺癌细胞系A549/PPAR-γ(-)，并通过RT-PCR和Western blot对构建结果进行验证。随后的MTT研究结果显示，A549/PPAR-γ(-)对顺铂的耐受性增加，表现为IC_50_值升高，并且耐受性与PPAR-γ的表达量成负相关，即PPAR-γ表达越低，耐受性越高。为了进一步地阐明PPAR-γ下调后赋予A549细胞耐药性的分子机制，根据已知的PPAR-γ在肿瘤细胞中可能发挥的作用，我们利用流式细胞术检测了肿瘤细胞的凋亡率。结果显示，肿瘤细胞对顺铂的耐受性提高与凋亡减少密切相关。PPAR-γ作为一个转录因子，本身无法直接对细胞凋亡产生影响，必须通过调控与凋亡相关的蛋白表达方能发挥作用。Bcl-2和caspase-3是一个在细胞凋亡中发挥重要作用的蛋白，在很多肿瘤中发现二者的表达与肿瘤的耐药密切相关^[17, 18]^。本研究发现，在A549/PPAR-γ(-)细胞中，bcl-2的蛋白表达水平明显升高，caspase-3表达明显下降，PPAR-γ表达下降致使bcl-2升高和caspase-3上调是肿瘤产生耐药的主要机制之一。进一步的RT-PCR证实，bcl-2的表达升高是通过bcl-2基因转录水平升高实现的，这与PPAR-γ作为转录因子的作用特点吻合。另外一个与肿瘤耐药密切相关的信号通路是PI3K/Akt^[5, 19]^。Western blot研究显示，A549/PPAR-γ(-)细胞中Akt的磷酸化水平升高，说明在该细胞中，PI3K/Akt的活化也参与到细胞对顺铂的耐药中，但PPAR-γ如何与PI3K/Akt进行对话尚不清楚，值得深入研究。上述这些调控均体现出与PPAR-γ表达量相关的效应关系。

总之，本研究显示沉默PPAR-γ可使A549细胞对顺铂的耐受性提高，其机制是通过上调bcl-2表达、增强PI3K/Akt信号通路，进而使得细胞抗药物诱导凋亡的能力增强。因此，PPAR-γ表达的下降很可能是临床肿瘤产生耐药的一种重要甚至是普遍的机制。同时，本研究不排除PPAR-γ还可能通过bcl-2和PI3K/Akt以外的途径增加肿瘤细胞的耐药性，值得进一步的研究。
